# A robust color image encryption algorithm based on 2D-SQSM hyperchaotic map and cyclic shift scrambling

**DOI:** 10.1371/journal.pone.0333640

**Published:** 2025-10-22

**Authors:** Feixian Liu, Shulei Wu

**Affiliations:** School of Information Science and Technology, Hainan Normal University, Haikou, Hainan, China; Balikesir Universitesi, TÜRKIYE

## Abstract

This paper proposes a structurally simplified 2D quadratic sine map (2D-SQSM). This map effectively addresses the insufficient chaos performance of traditional chaotic maps while avoiding the overly complex structures of emerging chaotic maps. Evaluated using multiple chaos performance metrics, the 2D-SQSM demonstrates high Lyapunov exponents, and sample entropy, with chaotic characteristics superior to some advanced chaotic maps proposed in recent years. Based on the 2D-SQSM, this paper further designs a highly robust color image encryption algorithm. First, by introducing different hash functions multiple times, the correlation between the key and plaintext is enhanced, significantly improving resistance against brute-force attacks; second, cyclic shifting and segmentation-recombination operations are applied separately to the three RGB channels to effectively disrupt pixel distribution and significantly reduce spatial correlation between pixels; finally, the chaotic sequence generated by the 2D-SQSM is utilized for XOR diffusion, further enhancing the randomness and diffusion capability of the ciphertext. A large number of simulation results demonstrate that this algorithm can significantly enhance the image information entropy, and can effectively reduce pixel correlation, possessing good statistical properties. Furthermore, it is robust against differential attacks, noise attacks, cropping attacks, chosen plaintext attacks, etc., and is suitable for secure image transmission.

## 1. Introduction

In today’s era, marked by technological and network advancements, images have become essential carriers of information. However, the widespread use and propagation of images also bring severe privacy and security challenges. As the threats of data breaches, cyberattacks, and unauthorized access become increasingly serious, it has become particularly important to ensure the security of digital images during storage, transmission, and retrieval [1].

Image encryption technology converts images into undecipherable cipher data through specific encryption means, preventing unauthorized accessors from obtaining valid information from them. Due to the characteristics of digital images, such as large amounts of information, strong pixel correlation, and statistical characteristics of pixel values, traditional text and data encryption schemes, such as AES, DES, are not suitable for image encryption [2].Therefore, researchers have discovered that chaos theory, as a mathematical tool for studying complex dynamic systems, shows broad application prospects in the field of information security due to its unique nonlinear and sensitive characteristics. The inherent stochastic properties and trajectory divergence characteristics of chaotic systems render them exceptionally suitable for cryptographic applications. Many researchers have also combined chaotic map with technologies such as DNA encoding [3–6], compressed sensing [7–9], quantum theory [10–12], neural networks [13–16], memristors [17–19], deep learning [20–22] and cellular automaton [23–25] to optimize the cryptographic security performance. However, these methods still face certain challenges: some schemes feature complex structures and high computational overhead, hindering real-time applications; DNA operations carry risks of implementation errors; and neural network models incur high training and deployment costs. Therefore, designing an encryption scheme that combines structural simplicity, high efficiency, and strong chaotic properties remains crucial for advancing the practical implementation of image encryption technology.

The robustness of visual data encryption schemes is fundamentally dependent on the dynamical characteristics of underlying chaotic mechanisms. Classical chaotic maps, often suffer from uneven trajectory distribution and discontinuous chaotic ranges. Multidimensional chaotic systems demonstrate significantly enhanced dynamical complexity and richer nonlinear characteristics when contrasted with their one-dimensional counterparts. However, if the dimension of the chaotic system is too large, such as reaching three-dimensional [26–28] or four-dimensional [29–31], it may lead to efficiency problems. Therefore, many scholars have proposed many two-dimensional maps through various methods. For example, Erkan U et al. [32] originally introduced a novel 2D chaotic map system constructed using the Schaffer function as its foundational framework.; Gao et al. [33] put forward a new type of two-dimensional extended Schaffer function map based on in-depth research on and inspiration from the Schaffer function. By making use of the unique chaotic characteristics of this new system and combining with advanced neural network technology, an algorithm specifically designed for encrypting the key regions of videos is designed. Numerous researchers have focused on enhancing the complexity of classical one-dimensional chaotic maps (e.g., Logistic, Sine, and Cubic) through nonlinear combination and function transformation. For example, Li et al. [34], Wang et al. [35], Lai et al. [36], Zhang et al. [37], Liu et al. [38], and Wang et al. [39] have respectively proposed composite or coupled map structures based on inverse trigonometric, exponential, logarithmic, sine, and cubic functions. By introducing various nonlinear terms for algebraic mixing or cascaded coupling, they aim to construct enhanced chaotic systems with a broader chaotic range, higher complexity, and improved randomness, thereby enhancing their application performance in domains such as image encryption. Although these maps have shown good chaotic performance, the structure is relatively complex and is not conducive to image encryption [40,41]. Therefore, after analyzing some classic chaotic maps, this paper proposes a two-dimensional simplified quadratic sine map by introducing quadratic terms and sine functions. The proposed 2D-SQSM demonstrates dual advantages over contemporary chaotic systems, combining structural simplicity with superior dynamical characteristics.

Furthermore, leveraging the 2D-SQSM framework, we develop a novel symmetric cryptographic scheme with enhanced security features. In the key generation part of the encryption algorithm, we apply different hash functions multiple times to increase the difficulty of brute-force cracking, and it is highly sensitive to plain images. To further enhance the security performance, a row-column transformation and circular shift scrambling algorithm based on RGB channels is introduced to scramble the pixel positions. Finally, use the chaotic sequences generated by 2D-SQSM to perform XOR operations with the scrambled image to further enhance the encryption effect. In conclusion, the following are the contributions and innovations of our work:

1. To address issues such as insufficient performance or complex structures in existing chaotic maps, a novel hyperchaotic map—2D-SQSM is proposed, which balances structural simplicity and complex chaotic characteristics.
2. Multiple performance evaluations demonstrate that 2D-SQSM outperforms various recently proposed chaotic maps in terms of chaotic behavior, exhibiting a wider chaotic range and stronger randomness.
3. Based on 2D-SQSM, a novel key generation mechanism is designed, and combined with cyclic shift and XOR diffusion strategies, an efficient and secure image encryption algorithm is developed.
4. Extensive simulation experiments and security analysis results indicate that the proposed algorithm performs excellently in resisting common attacks, offering both high security and practicality with strong application potential.

The structure of the remaining parts of this paper is arranged as follows: Part Two details the proposed 2D-SQSM chaotic map and analyzes its performance. Part Three describes the designed image encryption algorithm. Part Four and Part Five evaluate and compare the algorithm’s performance through simulation experiments and security analysis. Finally, Part Six is the summary.

## 2. Chaotic map

This section introduces the simplified two-dimensional quadratic sine map (2D-SQSM) proposed by us. To fully verify the superiority of the proposed map, we conduct performance tests based on multiple chaotic evaluation indicators and make a comparative analysis with several two-dimensional chaotic maps proposed in recent years.

### 2.1. Proposed chaotic map

To streamline the directly coupled two-dimensional map, after analyzing some classic chaotic maps, this paper constructs a two-dimensional simplified quadratic-sine map (2D-SQSM) by introducing a quadratic term and a sine function. Its mathematical expression is as follows:


$$\left\{ \begin{array}{c} x_{n+\text{1}}=(\alpha x_{n}^{\text{2}}+\text{1}\text{0}e^{\beta }\;\text{sin}\;y_{n})mod\text{1},\\ y_{n+\text{1}}=(\alpha y_{n}^{\text{2}}+\text{1}\text{0}e^{\beta }\;\text{sin}\;x_{n})mod\text{1}, \end{array}\right.\;$$ (1)

where $x_{n}$ and $y_{n}$ are the inputs for the map, while $x_{n+\text{1}}$ and $y_{n+\text{1}}$ are the corresponding outputs. α and β are parameters.

Compared with the other 6 chaotic maps listed in Table 1, 2D-SQSM has obvious advantages in terms of the simplicity of mathematical expressions, the simplification of control parameters, and scalability, etc.

**Table 1:** Six newly proposed chaotic maps.

Name	F(x,y)	Control parameters
2D-HELS [35]	$\left\{ \begin{array}{c} x_{n+\text{1}}=\text{4}\;\text{sin}\left(\pi (\text{4}e^{\mu }x_{n}(\text{1}-x_{n})+(\text{1}-e^{\mu }\text{sin}\left(\pi y_{n}\right))\right)\\ y_{n+\text{1}}=\text{4}\;\text{sin}\left(\pi (\text{4}e^{\mu }y_{n}(\text{1}-y_{n})+(\text{1}-e^{\mu })\;\text{sin}\left(\pi x_{n+\text{1}}\right))\right) \end{array}\right.\;$	μ
2D-LMHM [36]	$\left\{ \begin{array}{c} x_{n+\text{1}}=\beta (\text{2}\mu -\frac{x_{n}^{\text{2}}}{\mu })+k\;\text{sin}(y_{n})x_{n}\\ y_{n+\text{1}}=k_{\text{1}}x_{n}+k_{\text{2}}y_{n} \end{array}\right.\;$	β, μ, k, $k_{\text{1}}$, $k_{\text{2}}$
2D-ELSCM [37]	$\left\{ \begin{array}{c} x_{n+\text{1}}={\text{sin}}^{\text{2}}\left[m\pi^{\text{2}}(\text{ln}(x_{n})exp(y_{n})+\text{ln}\left(y_{n})exp(x_{n}\right))\right]\\ y_{n+\text{1}}={\text{sin}}^{\text{2}}\left[n\pi^{\text{2}}(\text{ln}\left(x_{n}y_{n})exp(x_{n}y_{n}\right))\right] \end{array}\right.\;$	m,n
2D-CLCM [38]	$\left\{ \begin{array}{c} x_{n+\text{1}}=mod(f(g(x_{n})+g(y_{n})),\text{1})\\ y_{n+\text{1}}=mod(g(f(x_{n})),\text{1}) \end{array}\right.\;$ $\left\{ \begin{array}{c} f(x_{n})=\text{4}ax_{n}(\text{1}-x_{n})\\ g(y_{n})=by_{n}(\text{1}-y_{n}^{\text{2}}) \end{array}\right.\;$	a, b
2D-NSLSLM [39]	$\left\{ \begin{array}{c} x_{n+\text{1}}=\text{sin}\left(\frac{\text{2}a\pi {\text{log}}_{\text{2}}(\text{1}+x_{n})(\text{1}-{\text{log}}_{\text{2}}\left(\text{1}+x_{n})\right)}{b{\text{tan}}^{-\text{1}}\left(\sqrt{\text{1}-y_{n}^{\text{2}}}\right)}\right)\;mod\text{1}\\ y_{n+\text{1}}=\text{sin}\left(\frac{\text{2}a\pi {\text{log}}_{\text{2}}(\text{1}+y_{n})(\text{1}-{\text{log}}_{\text{2}}\left(\text{1}+y_{n})\right)}{b{\text{tan}}^{-\text{1}}\left(\sqrt{\text{1}-x_{n}^{\text{2}}}\right)}\right)\;mod\text{1} \end{array}\right.\;$	a, b
2D-NHM [42]	$\left\{ \begin{array}{c} x_{n+\text{1}}=ax_{n}+b\;\text{cos}\left(\text{2}y_{n}\right)\\ y_{n+\text{1}}=cy_{n}+d\;\text{sin}\left(\text{2}x_{n}\right) \end{array}\right.\;$	a, b, c, d

### 2.2 Bifurcation and trajectory diagrams

**Fig 1:**
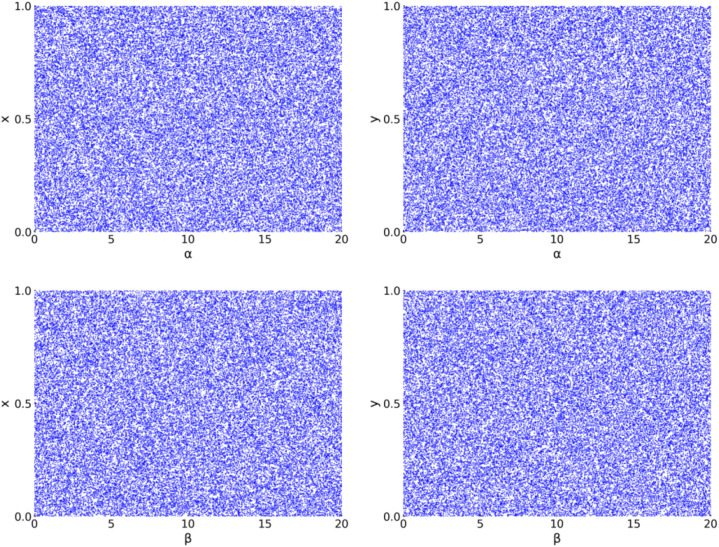
Bifurcation Diagrams of 2D-SQSM.

Bifurcation analysis and phase-space visualization provide insightful characterization of nonlinear dynamical systems. The bifurcation diagram quantitatively demonstrates how system states transition through periodic and chaotic regimes as control parameters vary, enabling precise identification of chaotic operating ranges. Fig 1 presents the bifurcation diagrams for both state variables (x,y) in the 2D-SQSM system, generated with initial conditions $\left(x_{\text{0}},y_{\text{0}}\right)$=(0.5, 0.6) and stsrting from parameters α=10, β=3, while varying control parameters (α,β) across the interval [0, 20]. The observed bifurcation patterns demonstrate that the system maintains robust chaotic behavior with spatially uniform distribution properties, independent of parameter variations.

**Fig 2:**
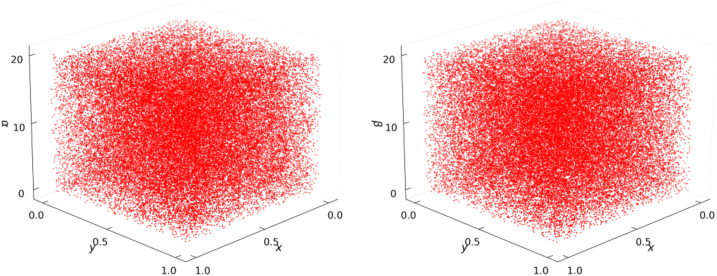
Trajectory Diagrams of 2D-SQSM.

The phase portrait analysis complements bifurcation studies by visualizing the system’s dynamical evolution in phase space. Fig 2 displays 3D trajectory plots of the 2D-SQSM system, revealing complete phase-space occupation with uniform stochastic distribution-a definitive manifestation of strong chaotic properties.

### 2.3. Lyapunov exponent

**Fig 3:**
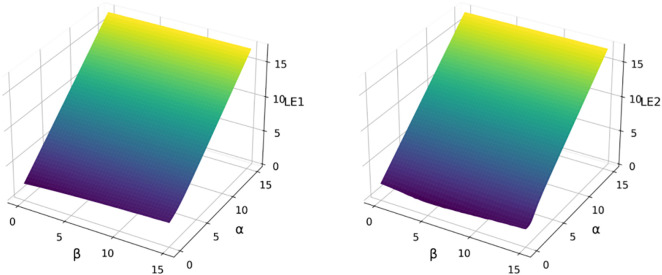
Lyapunov exponent diagrams of 2D-SQSM.

The Lyapunov exponent spectrum serves as a fundamental quantitative indicator for assessing chaotic dynamics, where the positivity of the maximal exponent in 2D systems constitutes a definitive criterion for chaos emergence. Fig 3 demonstrates that the 2D-SQSM’s Lyapunov exponents maintain persistent positivity across the parameter variation domain while exhibiting parameter-dependent monotonic growth, confirming the system’s robust chaotic regime.

**Fig 4:**
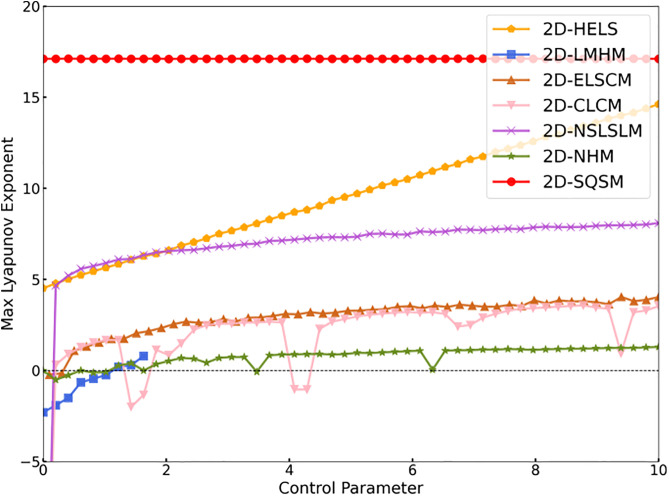
Comparison Graph of the Maximum Lyapunov Exponents between 2D-SQSM and Other Chaotic Maps.

It can be seen from Fig 4 and Table 2, through a comparative experiment with six other latest two-dimensional chaotic maps, the 2D-SQSM shows significant advantages: its maximum Lyapunov exponent not only has a larger value but also has a smaller fluctuation range. This result fully demonstrates that the 2D-SQSM has stronger sensitivity to initial values and better chaotic performance.

**Table 2:** The average values of the maximum lyapunov exponents, SE, and PE of 2D-SQSM and the Six Most Recently Proposed Chaotic Maps.

Name	Control parameters		${LE}_{max}$	${SE}_{x}$	${SE}_{y}$	${PE}_{x}$	${PE}_{y}$
	Fixed	Variable[0,10]					
2D-HELS		μ	9.6123	1.9280	1.9323	0.9983	0.9985
2D-LMHM	β=0.1, μ=100, $k_{\text{1}}$=1, $k_{\text{2}}$=0.1	k	-0.6426	0.0100	0.0103	0.1917	0.1871
2D-ELSCM	n=1	m	2.9659	1.8592	1.7942	0.9781	0.9769
2D-CLCM	b=3.99	a	1.8079	1.9275	2.0059	0.0684	0.0162
2D-NSLSLM	b=0.01	a	6.5551	2.0038	2.0089	0.9784	0.9780
2D-NHM	a=-1, c=0.65, d=0.9	b	0.7610	0.2729	1.1343	0.7795	0.9418
2D-SQSM	β=15	α	17.1153	2.1857	2.1844	0.9981	0.9978

### 2.4. Sample entropy

**Fig 5:**
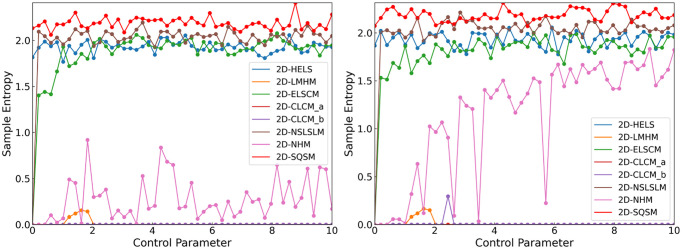
Comparison Graph of Sample Entropy between 2D-SQSM and Other Chaotic Maps.

Sample Entropy serves as a robust nonlinear dynamics measure for quantifying the structural complexity and pattern irregularity in temporal data sequences. Compared with the traditional Approximate Entropy, this metric has better statistical properties and anti-noise capabilities. As shown in Fig 5 and Table 2, through a comparative test of 2D-SQSM with six mainstream chaotic maps, the results show that two SE value of 2D-SQSM is significantly bigger than that of other comparison algorithms. The quantitative assessment conclusively establishes that the 2D-SQSM system generates chaotic sequences with superior complexity metrics compared to conventional approaches.

### 2.5. Permutation entropy

**Fig 6:**
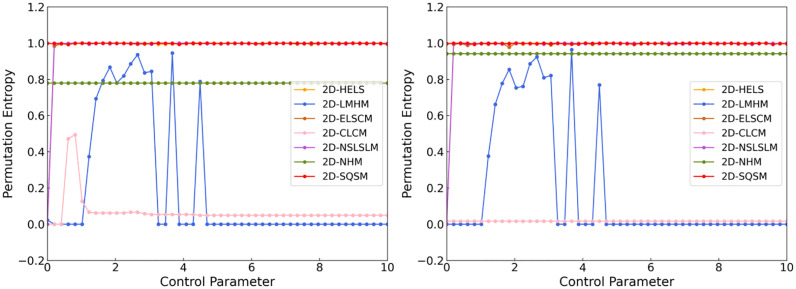
Comparison graph of permutation entropy between 2D-SQSM and other chaotic Maps.

Sample entropy quantifies the complexity of time series through sequence similarity under a given tolerance, while permutation entropy evaluates its irregularity by analyzing the order patterns of sequence elements. Fig 6 showcases a comparison graph of the permutation entropy. The results show that the entropy value of 2D-SQSM is close to 1, fully proving that this system has excellent sequence complexity and ideal stochastic characteristics.

### 2.6. NIST randomness test

The NIST test developed by the National Institute of Standards and Technology (NIST) is a standard method for comprehensively evaluating the randomness of sequences. To further prove that the 2D-SQSM has good randomness in a statistical sense, we conducted NIST randomness tests on the sequence generated by this chaotic map. Specifically, the system starts iterating with initial conditions $\left(x_{\text{0}},y_{\text{0}}\right)$=(0.5, 0.6) and control parameters α=8, β=10, generating a real-valued chaotic sequence of length 10,000,000. Subsequently, each chaotic state value x∈[0,1) is linearly mapped to the integer range [0, 255], producing a byte sequence, which is then post-processed by modular addition with random bytes to enhance randomness. Finally, the processed integer sequence is converted into a byte stream and saved as a binary file named output.bin, ready for reading and analysis by the NIST test suite. The individual bits in this file constitute the binary input sequence required for testing, thereby evaluating its statistical randomness.As shown in Table 3, all P-values are greater than the significance level of 0.01, indicating that this sequence has completely passed the test and has excellent random performance.

**Table 3:** NIST test result.

Test	P	Result
Frequency	0.9114	Pass
ApproximateEntropy	0.2133	Pass
BlockFrequency	0.7399	Pass
CumulativeSums	0.3505	Pass
FFT	0.7399	Pass
LinearComplexity	0.2133	Pass
LongestRun	0.0668	Pass
NonOverlappingTemplate	0.6371	Pass
OverlappingTemplate	0.7399	Pass
RandomExcursions	0.4372	Pass
RandomExcursionsVariant	0.1626	Pass
Rank	0.5341	Pass
Runs	0.2757	Pass
Serial	0.9643	Pass
Universal	0.2757	Pass

## 3. Encryption algorithm

This section introduces the encryption algorithm proposed by us based on the 2D-SQSM hyper-chaotic map. The algorithm is composed of three parts: key generation, pixel confusion, and XOR diffusion. We design the key generation method based on plaintext images to make the algorithm highly sensitive to plaintext; the cyclic shift operation is adopted to break the correlation between pixels; the XOR diffusion mechanism is introduced to further enhance the encryption effect and improve the overall security.

### 3.1. Algorithm overview

**Fig 7:**
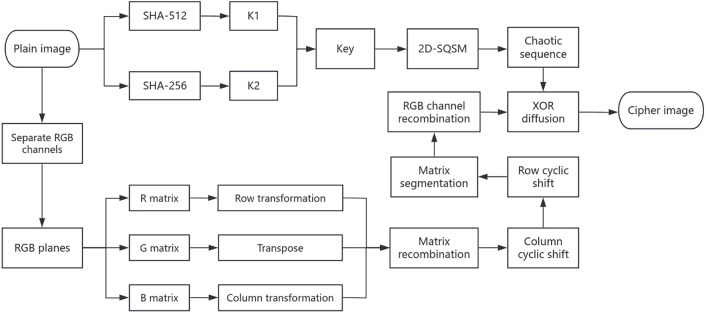
Image Encryption Flow Chart.

This research proposes a novel encryption algorithm for color images utilizing the 2D-SQSM chaotic system. First, the original image is processed using the dual hash algorithms of SHA-512 and SHA-256 to generate an initial key with plaintext-sensitive characteristics. Second, pixel-level spatial scrambling is achieved through RGB channel separation, matrix transformation (row/column transformation and transposition), and cyclic shift operations. Finally, the encrypted process is completed by performing pixel-by-pixel XOR diffusion between the normalized chaotic sequence generated by the 2D-SQSM and the scrambled image. As shown in Fig 7, this multi-level encryption architecture ensures that the encrypted image has excellent confidentiality and security through the synergistic effect of key generation, pixel obfuscation, and diffusion encryption.

### 3.2. Key generation method

The encryption proposal proposed in this paper uses plaintext images and SHA-512, SHA-256 hash algorithms to jointly generate a passkey. By applying different hash functions multiple times, it not only significantly increases the difficulty of violence cracking but also achieves high sensitivity to plaintext. The steps are as follows:

(1) Step 1: Initial Key Construction

- Input the plain image and extract its binary data.
- Generate a 512-bit master key $K_{\text{0}}$ using SHA-512 hashing.
- Split $K_{\text{0}}$ into two 256-bit subkeys $K_{\text{0}\text{1}}$ and $K_{\text{0}\text{2}}$.
- Compute the intermediate key $K_{\text{1}}$ via XOR operation: $K_{\text{1}}=K_{\text{0}\text{1}}⨁K_{\text{0}\text{2}}$.

(2) Step 2: Secondary Key Reinforcement

- Apply SHA-256 hashing to the plain image to produce a 256-bit auxiliary key $K_{\text{2}}$.

(3) Step 3: Final Key Derivation

- Generate the 256-bit final key K by mixing $K_{\text{1}}$ and $K_{\text{2}}$: $K=K_{\text{1}}⨁K_{\text{2}}$.

(4) Step 4: Parameter Initialization

- Partition K into four 64-bit segments $r_{\text{1}}$, $r_{\text{2}}$, $r_{\text{3}}$, and $r_{\text{4}}$, each represented as 16-digit hexadecimal values.
- Convert these segments into initial parameters $\left(x_{\text{0}},y_{\text{0}},\alpha ,\beta \right)$ for 2D-SQSM using the predefined map formula(2).

$$\left\{ \begin{array}{c} x_{\text{0}}=\frac{r_{\text{1}\_mean}-r_{\text{1}\_min}}{r_{\text{1}\_max}-r_{\text{1}\_min}}\times \text{2}-\text{1},\\ y_{\text{0}}=\frac{r_{\text{2}\_mean}-r_{\text{2}\_min}}{r_{\text{2}\_max}-r_{\text{2}\_min}}\times \text{2}-\text{1},\\ \alpha =\frac{r_{\text{3}\_mean}-r_{\text{3}\_min}}{r_{\text{3}\_max}-r_{\text{3}\_min}}\times \text{1}\text{0},\\ \beta =\frac{r_{\text{4}\_mean}-r_{\text{4}\_min}}{r_{\text{4}\_max}-r_{\text{4}\_min}}\times \text{1}\text{0}, \end{array}\right.\;$$ (2)

where $r_{i\_max}$, $r_{i\_min}$, and $r_{i\_mean}$ are the maximum, minimum, values of the 16 hexadecimal strings in $r_{i}$, respectively.

**Algorithm 1** Key Generation

**Input:** Image P.

1: $K_{\text{0}}=SHA-\text{512}(P);$

2: $K_{\text{01}}=K_{\text{0}}[\text{0}:\text{255}],K_{\text{02}}=K_{\text{0}}[\text{256}:\text{511}];$

3: $K_{\text{1}}=K_{\text{01}}⨁K_{\text{02}};$

4: $K_{\text{2}}=SHA-\text{256}(P);$

5: $K=K_{\text{1}}⨁K_{\text{2}};$

6: $r_{\text{1}},r_{\text{2}},r_{\text{3}},r_{\text{4}}=split(K);$

7: $x_{\text{0}}=\frac{r_{\text{1}\_mean}-r_{\text{1}\_min}}{r_{\text{1}\_max}-r_{\text{1}\_min}}\times \text{2}-\text{1}$;$y_{\text{0}}=\frac{r_{\text{2}\_mean}-r_{\text{2}\_min}}{r_{\text{2}\_max}-r_{\text{2}\_min}}\times \text{2}-\text{1}$;

     $\alpha =\frac{r_{\text{3}\_mean}-r_{\text{3}\_min}}{r_{\text{3}\_max}-r_{\text{3}\_min}}\times \text{10}$;$\beta =\frac{r_{\text{4}\_mean}-r_{\text{4}\_min}}{r_{\text{4}\_max}-r_{\text{4}\_min}}\times \text{10}$.

**Output:**$\left(x_{\text{0}},y_{\text{0}},\alpha ,\beta \right)$.

### 3.3. Cyclic scrambling

The scrambling algorithm used in this paper first separates the three RGB channels of the plain image, and sequentially performs row transformation, column transformation, and transpose operations on the matrix of each channel to construct a comprehensive matrix. Subsequently, the positions of row and column pixels in the comprehensive matrix are deeply scrambled through cyclic shifting. Finally, the scrambled RGB image is obtained through block recombination operations. The steps are as follows:

(1) Step1: Separate the input plain image into three color channels of RGB, forming three matrices R, G, and B of size n×n.
(2) Step2: For the input matrix R, construct matrix $R_{1}$ through row transformation, and rearrange the elements in its i-th row to the position of the (n−i+1)-th row.
(3) Step3: Calculate the transpose of matrix G as matrix $G_{1}$, i.e.,$G_{\text{1}}(i,j)=G(j,i)$.
(4) Step4: For the input matrix B, construct matrix $B_{1}$ through column transformation, and rearrange the elements in its i-th column to the position of the (n−i+1)-th column.
(5) Step5: Matrix C is constructed using row interleaving technique, where its (3i−2)-th, (3i−1)-th, and 3i-th rows are taken from the i-th rows of matrices $R_{1}$, $G_{1}$, and $B_{1}$, respectively.
(6) Step6: Circular shift: Perform a cyclic shift at the ${\left(-\text{1}\right)}^{j-\text{1}}V(j)$ position on each column of matrix C to obtain $C_{1}$. The random integer array $V\in Z^{n}$ is used to decide the cyclic shift amount for each column. Assume that each component in V is 1.
(7) Step7: Perform a cyclic shift at position ${\left(-\text{1}\right)}^{i-\text{1}}W(i)$ for each row of matrix $C_{1}$ to obtain $C_{2}$. The random integer array $W\in Z^{\text{3}n}$ is used to decide the cyclic shift amount for each row. Assume that each component in W is 1.
(8) Step8: Split the rows of matrix $C_{2}$ to construct three matrices. The first n rows of matrix $C_{2}$ form matrix $R^{\prime }$, the middle n rows form matrix $G^{\prime }$, and the last n rows form matrix $B^{\prime }$.
(9) Step9: Combine the three rearranged matrices $R^{\prime }$, $G^{\prime }$, and $B^{\prime }$ back into a complete RGB image.

**Algorithm 2** Circular Scrambling

**Input:** RGB image M of size n×n×3.

1: R,G,B=split(M);

2: $R_{1}(i,:)=R(n-i+\text{1},:\forall i\in [\text{1},n];$

3: $G_{1}=G^{𝐓};$

4: $B_{1}(:,j)=B(:,n-j+\text{1}\forall j\in [\text{1},n];$

5: $C(\text{3}i-\text{2},:)=R_{1}(i,:),C(\text{3}i-\text{1},:)=G_{1}(i,:),C(\text{3}i,:)=B_{1}(i,:);$

6: $C_{1}(:,j)=circshift(C(:,j),{-\text{1}}^{j-\text{1}}v_{j}\;\forall j\in [\text{1},n];$

7: $C_{2}(i,:)=circshift(C_{1}(i,:),{-\text{1}}^{i-\text{1}}w_{j}\;\forall i\in [\text{1},\text{3}n];$

8: $R^{\prime }=C_{2}[\text{1}:n,:],G^{\prime }=C_{2}[n+\text{1}:\text{2}n,:],B^{\prime }=C_{2}[\text{2}n+\text{1}:\text{3}n,:];$

9: $M^{\prime }=merge(R^{\prime },G^{\prime },B^{\prime })$.

**Output:** Scrambled image $M^{\prime }$.

### 3.4. XOR diffusion

The diffusion mechanism capitalizes on the intrinsic nonlinear dynamics and trajectory divergence properties of chaotic systems to strengthen encryption robustness. In our proposed encryption algorithm, the diffusion phase is implemented through the following steps:

(1) Step1: Four initial states $\left(x_{\text{0}},\;y_{\text{0}},\alpha ,\beta \right)$ of 2D-SQSM determined by key K, and discarding the first $N_{\text{0}}$ values, generate a pseudo-random sequence whose length corresponds precisely to the pixels count of input image through 2D-SQSM.
(2) Step2: The two-dimensional image array undergoes vectorization transformation to enable element-wise diffusion via bitwise XOR processing with the pseudo-random chaotic sequence.
(3) Step3: Normalize the generated chaotic sequence to the pixel intensity range of 0–255.
(4) Step4: Each original pixel value undergoes bitwise XOR transformation with its corresponding pseudo-random sequence element to generate the diffused cipher pixels.

**Algorithm 3** Diffusion

**Input:** Image Q, $\left(x_{\text{0}},y_{\text{0}},\alpha ,\beta \right)$.

1: $S=\text{2D}-\text{SQSM}(x_{\text{0}},y_{\text{0}},\alpha ,\beta )$;

2: $S=S[N_{\text{0}}+\text{1}:];$

3: $S^{\prime }=\left\lfloor \text{255}\cdot S\right\rfloor ;$

4: $Q_{vec}=reshape(Q),S_{vec}=reshape(S^{\prime });$

5: $C_{vec}=Q_{vec}⨁S_{vec};$

6: $C=reshape(C_{vec}).$

**Output:** C.

### 3.5. Encryption steps

Assume that the size of the input color image P is N × N × 3. The specific encryption steps are as follows:

(1) Step1: Read the color image P as the raw input of the encryption system.
(2) Step2: Based on the scrambling algorithm proposed in Section 3.3, perform spatial position permutation on the three RGB channels of image P respectively to generate the intermediate cipher image Q.
(3) Step3: Use the key derivation scheme in Section 3.2 to generate the initial parameters of the 2D-SQSM chaotic map, and these parameters will be used as the diffusion keys for the entire encryption process.
(4) Step:4: After performing bit-plane shifting on the scrambled image Q, using the chaotic sequence generated by 2D-SQSM, the pixel value transformation is completed according to the diffusion algorithm in Section 3.4.Finally generate the encrypted image C.

Note: Decryption is the inverse operation of encryption.

## 4. Simulation experiments and results

**Fig 8:**
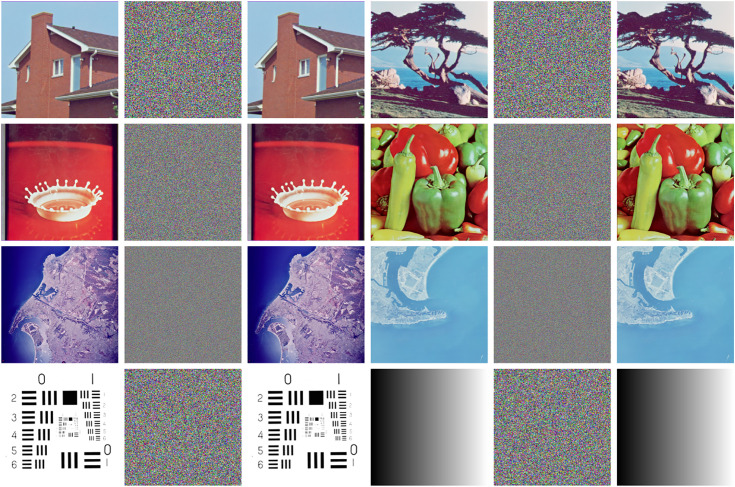
Encrypted and decrypted images.

The experimental environment is configured with an Intel(R) Core(TM) i5-8265U processor, 16GB of memory, Windows 10 operating system, and Python 3.12.4 development environment. The cryptographic transformation results are visually demonstrated in Fig 8. Take the pictures House, Tree(256 × 256 × 3), Splash, Peppers(512 × 512 × 3), San Diego(1024 × 1024 × 3), Resolution chart, Gradient(256 × 256) as examples.

## 5. Security analysis

### 5.1. Key space analysis

Among the various types of attacks, the brute-force attack is the most fundamental and widely used. Attackers implement the attack by systematically attempting every possible key within the key space [41]. Therefore, to ensure adequate resistance against brute-force attacks, the key space of the proposed IE algorithm should be no smaller than 2^128^ [43,44]. In the encryption algorithm, we set the precisions of $x_{\text{0}}$, $y_{\text{0}}$, α, and β as 10^-15^, 10^-15^, 10^-14^, and 10^-14^ respectively. Thus, we can calculate that the size of the key space is 10^15^ × 10^15^ × 10^14^ × 10^14^ ≈ 2^193^, which is far larger than the security threshold required in cryptography. Such a large key space makes it difficult to traverse all possible key combinations within a limited time even if modern supercomputers are used for brute-force attacks, thus providing sufficient security guarantees for the system.

### 5.2. Key sensitivity analysis

**Fig 9:**
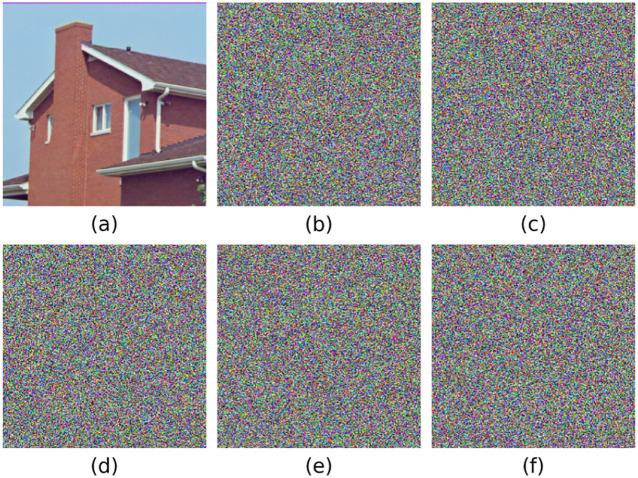
Comparison of the Decryption Effect between the Correct Key and the Key after Interference is Added. **(a) Plain Image; (b) Cipher Image; (c)**${𝐱}_{0}=x_{0}+10^{-15}$; **(d)**${𝐲}_{0}=y_{0}+10^{-15}$; **(e)**$\alpha =\alpha +10^{-15}$; **(f)**$\beta =\beta +10^{-15}$.


Key sensitivity serves as a fundamental security criterion for cryptographic systems, quantifying the algorithm’s differential response to minimal key variations. We have conducted further analysis and selected a small perturbation range of $\Delta x={\text{10}}^{-\text{15}}$ for sensitivity testing on each key parameter (including $x_{\text{0}},y_{\text{0}},\alpha ,\beta$). Take the House image of 265 × 265 as an example. Fig 9 presents a comparative visualization of decryption outcomes. Specifically, in our experiments, we applied this perturbation to each of the aforementioned parameters individually and used Mean Squared Error (MSE) as the evaluation metric to quantify how these parameter changes affect the model output. The calculation formula is:

$$\text{MSE}=\frac{\text{1}}{MN}\sum_{i=\text{1}}^{M}\sum_{j=\text{1}}^{N}{\left[I_{\text{1}}(i,\text{j})-I_{\text{2}}(i,j)\right]}^{\text{2}},$$ (3)

where $I_{\text{1}}$ and $I_{\text{2}}$ are two images, M and N are the height and width of the images respectively, and i and j are the index positions of the pixels. It can be seen from Table 4 that under a perturbation of ${\text{10}}^{-\text{15}}$, the average value of the MSE between the decrypted image and the encrypted image is 105.5888, which indicates that the algorithm has key sensitivity.

**Table 4:** MSE between the Cipher Image and the Decrypted Image under Different Parameter Perturbations.

	$x_{\text{0}}$	$y_{\text{0}}$	α	β
MSE	105.5147	105.5144	105.7252	105.6008

### 5.3. Histogram analysis

**Fig 10:**
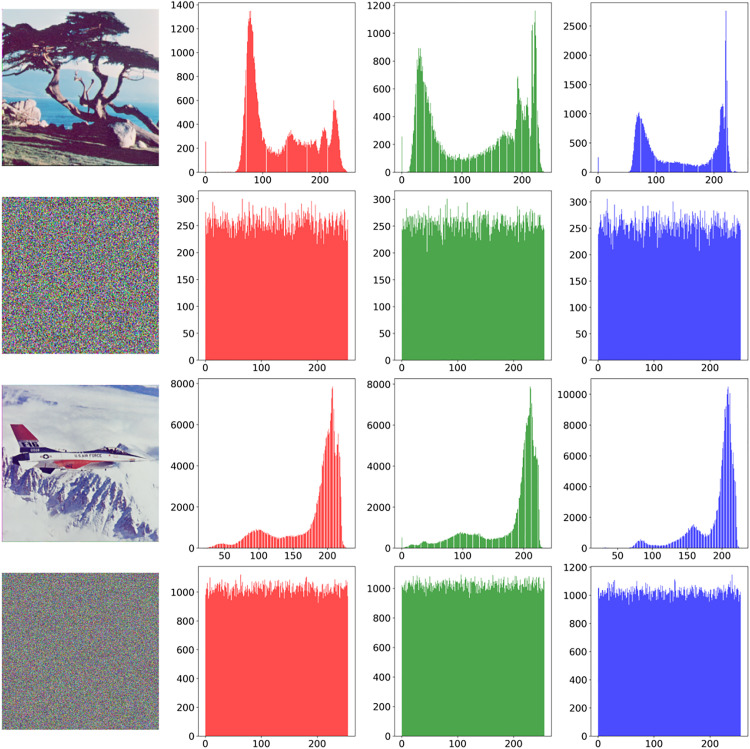
Pixel Histogram of Plain Image and Cipher Image of Tree and Airplane.

Histogram evaluation serves as a critical cryptographic assessment tool for analyzing pixel value distribution characteristics in encrypted images. This analytical approach primarily examines the statistical transformation of pixel value distributions between plain and cipher images. The pixel values of natural images usually exhibit non-uniform distribution characteristics, with the frequency of certain gray levels being significantly higher than that of other values. An optimal encryption scheme should produce cipher-images with statistically flat histograms, where all grayscale values demonstrate approximately equal. Taking the Tree(265 × 265) and Airplane(512 × 512) images as examples. Fig 10 presents comparative histogram analyses across RGB channels, distinctly revealing the plaintext images’ characteristic non-uniform pixel distributions versus the ciphertext’s quasi-uniform frequency patterns. After encryption processing, the pixel values in the three channels all change to an approximately uniform distribution, and the differences in the frequencies of occurrence of each gray level are significantly reduced. These findings demonstrate that the proposed cryptographic scheme successfully obliterates the original image’s statistical fingerprints while satisfying cryptographically rigorous randomness criteria for cipher.

### 5.4. Correlation analysis

**Fig 11:**
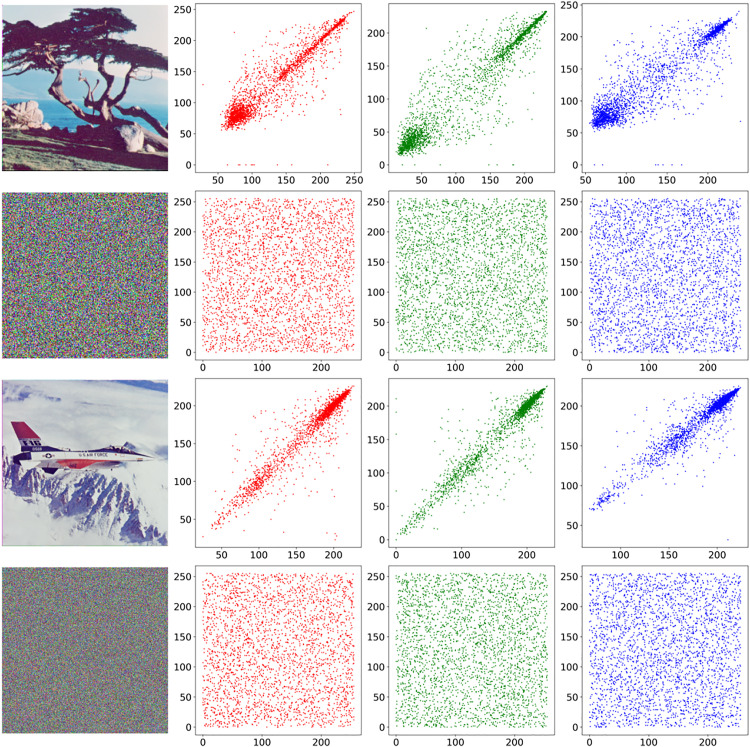
Pixel correlation graph of plain image and cipher image of tree and airplane.


Natural images inherently exhibit strong inter-pixel spatial dependencies. A robust encryption scheme must effectively disrupt these correlational patterns to ensure cryptographic security. We used the correlation coefficient defined by formula (4) to conduct a comparative analysis of pixel correlation before and after encryption on two test images, Tree(265 × 265) and Airplane(512 × 512). The comparative analysis in Fig 11 shows: the original image shows significant linear correlation; while the pixel distribution of the encrypted image shows a uniform scatter feature, and the correlation coefficient drops to an ideal value close to 0. Table 5 compares the changes in pixel correlation of nine color images before and after encryption. This result indicates that the algorithm successfully destroys the inherent spatial correlation characteristics of the image.

**Table 5:** Comparison of Pixel Correlation Before and After Encryption.

Image		Plain Image			Cipher Image		
		R	G	B	R	G	B
4.1.01 256 × 256	H	0.9729	0.9719	0.9584	−0.0031	0.0009	0.0021
	V	0.9622	0.9647	0.9519	0.0083	0.0026	0.0026
	D	0.9482	0.9500	0.9377	0.0007	−0.0005	−0.0031
4.1.02	H	0.9493	0.9308	0.9178	0.0001	−0.0010	−0.0002
	V	0.9562	0.9534	0.9442	0.0043	−0.0010	−0.0021
	D	0.9176	0.9002	0.8890	−0.0001	0.0005	0.0085
4.1.03	H	0.9779	0.9748	0.9726	0.0014	−0.0088	0.0035
	V	0.9294	0.9106	0.9130	−0.0077	0.0031	−0.0028
	D	0.9129	0.8941	0.8958	−0.0040	0.0018	0.0017
4.1.04	H	0.9786	0.9660	0.9523	0.0026	0.0023	0.0008
	V	0.9879	0.9820	0.9718	0.0020	−0.0043	−0.0037
	D	0.9684	0.9507	0.9306	−0.0079	0.0023	0.0024
4.2.01 512 × 512	H	0.9936	0.9812	0.9826	0.0005	0.0008	−0.0005
	V	0.9951	0.9871	0.9789	−0.0035	−0.0012	0.0005
	D	0.9894	0.9711	0.9649	0.0030	0.0010	0.0027
4.2.03	H	0.9231	0.8655	0.9073	0.0024	0.0021	0.0022
	V	0.8660	0.7650	0.8809	0.0017	−0.0024	−0.0001
	D	0.8543	0.7348	0.8399	0.0005	0.0021	0.0024
4.2.05	H	0.9726	0.9578	0.9640	−0.0017	0.0005	0.0024
	V	0.9568	0.9678	0.9353	−0.0007	0.0002	−0.0004
	D	0.9343	0.9326	0.9146	−0.0037	−0.0032	0.0032
4.2.06	H	0.9558	0.9715	0.9710	−0.0003	−0.0024	0.0004
	V	0.9541	0.9663	0.9694	−0.0003	−0.0029	0.0033
	D	0.9420	0.9530	0.9530	−0.0001	−0.0019	0.0019
4.2.07	H	0.9635	0.9811	0.9665	0.0015	−0.0019	0.0044
	V	0.9663	0.9818	0.9664	0.0006	0.0009	0.0021
	D	0.9564	0.9687	0.9478	0.0021	0.0011	0.0029
2.2.01 1024 × 1024	H	0.9260	0.9182	0.9077	0.0012	−0.0006	−0.0022
	V	0.9230	0.9153	0.9045	−0.0009	0.0004	−0.0012
	D	0.9043	0.8957	0.8838	0.0018	0.0001	0.0008
2.2.02	H	0.9315	0.8814	0.7899	0.0014	−0.0003	−0.0002
	V	0.9315	0.8820	0.7889	−0.0005	−0.0002	0.0017
	D	0.9046	0.8406	0.7392	−0.0012	0.0006	−0.0008

$$\left\{ \begin{array}{c} R_{xy}=\frac{cov(x,y)}{\sqrt{D(x)}\sqrt{D(y)}},\\ E(x)=\frac{\text{1}}{N}\sum_{i=\text{1}}^{N}x_{i},\\ D(x)=\frac{\text{1}}{N}\sum_{i=\text{1}}^{N}{\left(x_{i}-E(x)\right)}^{\text{2}},\\ cov(x,y)=\frac{\text{1}}{N}\sum_{i=\text{1}}^{N}\left(x_{i}-E(x))(y_{i}-E(y)\right), \end{array}\right.\;$$ (4)

where x and y are two data sequences, N is the total number of data points, and $x_{i}$ and $y_{i}$ represent the individual data points at the i-th position in these sequences, respectively.

### 5.5. Information entropy analysis

Developed by Claude Shannon, information entropy serves as a fundamental quantitative measure for assessing data unpredictability. Within cryptographic image processing, this metric precisely characterizes pixel value stochasticity—elevated entropy levels correspond to enhanced pixel uniformity and maximized ciphertext indeterminacy. For a discrete random variable X, the mathematical definition of its information entropy H(X) is:


$$H(X)=-\sum_{i=\text{1}}^{\text{n}}\text{p}\left(x_{i}\right)\;{\text{log}}_{\text{2}}\;\text{p}\;\left(x_{i}\right),$$ (5)

where $\text{p}(x_{i})$ represents the probability that the random variable X takes the value $x_{i}$, and n is the total number of all possible values.

Table 6 presents a comparison of entropy values achieved by various encryption techniques. The results indicate that the entropy values of the images encrypted using the proposed method are very close to the theoretical maximum of 8, outperforming existing approaches, which shows that this algorithm can generate a highly uniform pixel distribution and has excellent randomization performance.

**Table 6:** Comparison of Entropy of Cipher Images for Different Encryption Methods.

Image		Proposed	Ref. [41]	Ref. [45]	Ref. [46]	Ref. [47]	Ref. [48]	Ref. [49]
4.1.01 256 × 256	R	7.9976		7.9975	7.9967	——	7.9973	7.9971
	G	7.9973	——	7.9978	7.9971		7.9972	7.9972
	B	7.9973		7.9969	7.9973		7.9972	7.9967
4.1.02	R	7.9975		——	7.9968	——	7.9970	——
	G	7.9973	——		7.9976		7.9971	
	B	7.9972			7.9969		7.9970	
4.1.03	R	7.9971		7.9971	7.9971	——	7.9973	7.9969
	G	7.9971	——	7.9970	7.9974		7.9971	7.9972
	B	7.9975		7.9973	7.9973		7.9970	7.997
4.1.04	R	7.9975	7.9971	——	7.9970	——	7.9971	——
	G	7.9972	7.9972		7.9976		7.9974	
	B	7.9975	7.9972		7.9972		7.9973	
4.2.01 512 × 512	R	7.9993		7.9994	7.9993	——	7.9993	7.9993
	G	7.9993	——	7.9993	7.9992		7.9993	7.9994
	B	7.9993		7.9992	7.9993		7.9993	7.9994
4.2.03	R	7.9993		7.9993	7.9993	7.9972	7.9993	7.9993
	G	7.9994	——	7.9992	7.9992	7.9973	7.9993	7.9993
	B	7.9992		7.9992	7.9993	7.9970	7.9992	7.9993
4.2.05	R	7.9992		7.9993	7.9993	7.9971	7.9992	7.9993
	G	7.9994	——	7.9993	7.9993	7.9971	7.9993	7.9992
	B	7.9992		7.9993	7.9993	7.9973	7.9993	7.9994
4.2.06	R	7.9993		7.9992	——	7.9973	7.9993	——
	G	7.9993	——	7.9993		7.9971	7.9992	
	B	7.9993		7.9994		7.9969	7.9993	
4.2.07	R	7.9993		7.9991	——	7.9969	7.9993	7.9993
	G	7.9993	——	7.9992		7.9973	7.9993	7.9992
	B	7.9994		7.9994		7.9970	7.9992	7.9992
2.2.02 1024 × 1024	R	7.9998	7.9998					
	G	7.9998	7.9998	——	——	——	——	——
	B	7.9998	7.9998					

### 5.6. Differential analysis

**Fig 12:**
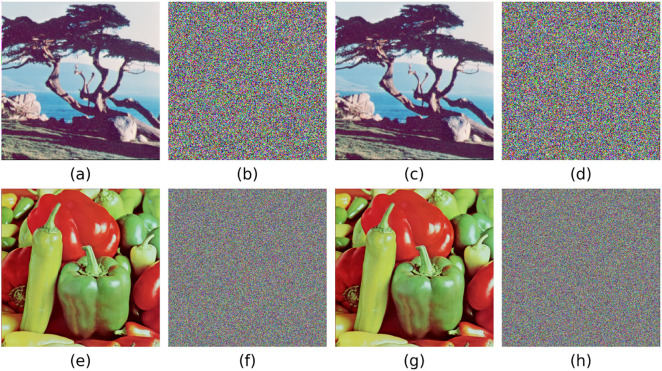
Results of Differential Attack Experiment. (a) Female; (b) Cipher Image of (a); (c) Female Image with One Pixel Change; (d) Cipher Image of (c); (e) Peppers; (f) Cipher Image of (e); (g) Peppers Image with One Pixel Change; (h) Cipher Image of (g).

Differential cryptanalysis is a method of cryptanalysis, where attackers analyze the impact of subtle changes in plain images on cipher to reveal the potential correlation between plain and cipher. To effectively withstand differential attacks, even minor alterations in the original image should lead to substantial variations in the resulting encrypted images. Fig 12 shows the original images of Tree(256 × 256) and Peppers(512 × 512) and their cipher images after modifying one pixel respectively. It can be seen that even if only one pixel bit is changed, almost completely different cipher images will be obtained.

To evaluate how effectively cryptographic algorithms can withstand differential attacks, two quantitative measures—specifically, the Number of Pixel Change Rate (NPCR) and the Unified Average Change Intensity (UACI)—are employed for performance analysis. The calculation formula is as follows:


$$\text{NPCR}=\frac{\sum_{i,j}D(i,j)}{M*N}*\text{1}\text{0}\text{0}\%,$$ (6)

$$\text{NPCR}=\frac{\sum_{i,j}D(i,j)}{M*N}*\text{1}\text{0}\text{0}\%,$$ (7)

$$\text{UACI}=\frac{\text{1}}{M*N}\frac{\sum \left(C_{\text{1}}(i,j)-C_{\text{2}}(i,j)\right)}{\text{2}\text{5}\text{5}}*\text{1}\text{0}\text{0}\%,$$ (8)

where M and N respectively represent the width and height of the image, and $C_{\text{1}}$ and $C_{\text{2}}$ are two cipher images. As shown in Tables 7 and 8, the NPCR and UACI values of this algorithm are both closest to the ideal values (NPCR: 99.6093%, UACI: 33.4635%), showing stronger resistance to differential attacks compared with other algorithms.

**Table 7:** Comparison of NPCR among different algorithms.

Image		Proposed	Ref. [45]	Ref. [50]	Ref. [46]	Ref. [51]	Ref. [52]	Ref. [49]
4.1.01 256 × 256	R	99.6077	99.6033	——	99.6346	——	99.6078	——
	G	99.5958	99.6159		99.6340		99.5926	
	B	99.6124	99.6196		99.6337		99.6063	
4.1.02	R	99.6124	——	——	99.6205	——	——	——
	G	99.6170			99.6200			
	B	99.5850			99.6197			
4.1.03	R	99.6277	99.5998	——	99.6399	——	99.6368	——
	G	99.5819	99.6071		99.6397		99.5987	
	B	99.6048	99.6115		99.6390		99.6399	
4.1.04	R	99.6033	——	——	99.6323	——	——	——
	G	99.6124			99.6318			
	B	99.6082			99.6314			
4.2.01 512 × 512	R	99.6103	99.6136	——	99.6201	——	99.6086	99.6087 99.6112 99.6050
	G	99.6010	99.6083		99.6199		99.6006	
	B	99.6010	99.6116		99.6202		99.6109	
4.2.03	R	99.6178	99.6039	99.6197	99.6390	99.6503	99.6063	99.6045
	G	99.6082	99.6064	99.6181	99.6388	99.6539	99.6086	99.6108
	B	99.6246	99.6046	99.6250	99.6389	99.6347	99.6086	99.6191
4.2.05	R	99.6109	99.6129	99.6185	99.6137	——	99.6052	99.6056
	G	99.5908	99.6073	99.6125	99.6135		99.5956	99.6116
	B	99.6166	99.6101	99.6113	99.6137		99.6143	99.6138
4.2.06	R	99.6147	99.6089	99.6173	——	——	——	99.6112
	G	99.6174	99.6049	99.6372				99.6070
	B	99.6197	99.6108	99.6254				99.6061
4.2.07	R	99.6069	99.6112	99.6231	——	99.6799	99.6006 99.6082 99.6021	99.6080
	G	99.6117	99.6086	99.6098		99.6131		99.6069
	B	99.6174	99.6096	99.6147		99.6988		99.6041

**Table 8:** Comparison of UACI among different algorithms.

Image		Proposed	Ref. [45]	Ref. [50]	Ref. [46]	Ref. [51]	Ref. [52]	Ref. [49]
4.1.01 256 × 256	R	33.4557	33.4649	——	33.4779	——	33.5234	——
	G	33.4634	33.4120		33.4545		33.2569	
	B	33.4775	33.4432		33.4497		33.343	
4.1.02	R	33.3759	——	——	33.4505	——	——	——
	G	33.4192			33.4082			
	B	33.2857			33.4788			
4.1.03	R	33.4552	33.4942	——	33.4902	——	33.5643	——
	G	33.4743	33.4994		33.4655		33.3784	
	B	33.4696	33.4725		33.4955		33.5677	
4.1.04	R	33.4006	——	——	33.4606	——	——	——
	G	33.3418			33.4319			
	B	33.4659			33.5558			
4.2.01 512 × 512	R	33.4770	33.4677	——	33.5286	——	33.406	33.5129
	G	33.4544	33.4563		33.5382		33.4824	33.4478
	B	33.4645	33.4619		33.5370		33.466	33.4273
4.2.03	R	33.5012	33.4903	33.4954	33.4753	33.4870	33.4551	33.4655
	G	33.4981	33.4398	33.4996	33.4374	33.4247	33.4352	33.4101
	B	33.5273	33.4451	33.5360	33.4417	33.4496	33.4769	33.4822
4.2.05	R	33.4479	33.4654	33.4845	33.4311	——	33.4651	33.4938
	G	33.4570	33.4373	33.5153	33.4208		33.534	33.4756
	B	33.4654	33.4563	33.5251	33.4145		33.3952	33.4568
4.2.06	R	33.4662	33.4819	33.5043	——	——	——	33.4386
	G	33.4603	33.4343	33.4647				33.4555
	B	33.5027	33.4448	33.4767				33.4438
4.2.07	R	33.4680	33.4599	33.5705	——	33.5543	33.5401 33.4724 33.3762	33.4564
	G	33.4449	33.4666	33.4751		33.4299		33.4727
	B	33.4770	33.4612	33.5267		33.4694		33.4897

### 5.7 Anti-noise attack

**Fig 13:**
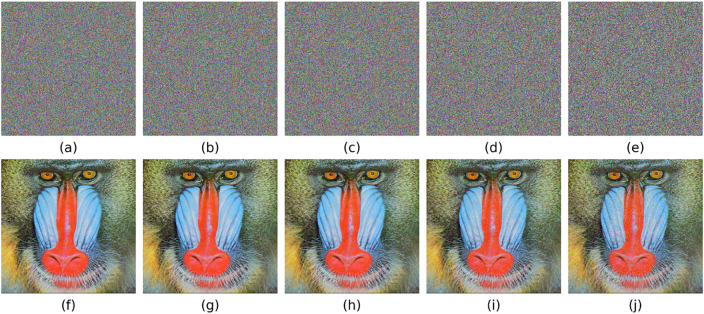
Robustness of the Encryption Algorithm Against Noise Pollution. (a) ,(b),(c),(d),(e) Cipher Image with 0.1%, 0.5%, 1%, 5%, 10% Salt and Pepper Noise; (f) ,(g),(h),(i),(j) Decrypted Image of (a) ,(b),(c),(d),(e).

Encrypted images may be interfered by noise during transmission. Therefore, an excellent encryption algorithm needs to have the ability to resist noise pollution. To verify this characteristic, this study selects the Baboon(512 × 512) image. After adding 0.1%, 0.5%, 1%, 5%, and 10% salt-and-pepper noise to their ciphertexts respectively, decryption experiments are conducted. We adopted Peak Signal-to-Noise Ratio (PSNR) and Structural Similarity Index Measure (SSIM) as metrics to evaluate the quality of decrypted images. Their calculation formulas are respectively:


$$\text{PSNR}=\text{10}\cdot {\text{log}}_{\text{10}}\left(\frac{{\text{MAX}}^{\text{2}}}{\text{MSE}}\right),$$ (9)

among them, MAX is the possible maximum pixel value in the image, and MSE is the mean square error.


$$\left\{ \begin{array}{c} \text{SSIM}=\frac{\left(\text{2}\mu_{x}\mu_{y}+C_{\text{1}})(\text{2}\sigma_{xy}+C_{\text{2}}\right)}{\left(\mu_{x}^{\text{2}}+\mu_{y}^{\text{2}}+C_{\text{1}})(\sigma_{x}^{\text{2}}+\sigma_{y}^{\text{2}}+C_{\text{2}}\right)},\\ C_{\text{1}}={\left(K_{\text{1}}L\right)}^{\text{2}},\\ C_{\text{2}}={\left(K_{\text{2}}L\right)}^{\text{2}}, \end{array}\right.\;$$ (10)

where $\mu_{x}$ and $\mu_{y}$, $\sigma_{x}^{\text{2}}$ and $\sigma_{y}^{\text{2}}$, $\sigma_{xy}$ are respectively the average value, variance, and covariance of images x and y within the local window, L is the dynamic range of pixels, and usually $K_{\text{1}}=\text{0}.\text{0}\text{1},K_{\text{2}}=\text{0}.\text{0}\text{3}$. Fig 13 demonstrate that even when subjected to strong noise interference (5%、10%), the decrypted images remain highly recognizable with minimal degradation in visual quality. Table 9 shows that even when subjected to noise attacks, the decrypted image can still maintain high PSNR and SSIM. This indicates that the proposed algorithm exhibits strong robustness against noise attacks.

**Table 9:** PNSR and SSIM under different noise attacks.

Image		Noise intensity				
		0.1%	0.5%	1%	5%	10%
Baboon	PSNR	38.23	31.78	28.81	21.87	18.82
	SSIM	0.9956	0.9811	0.9636	0.8410	0.7208
[53]	PSNR	——	——	27.98	——	17.98

### 5.8. Resistance to cropping attacks

**Fig 14:**
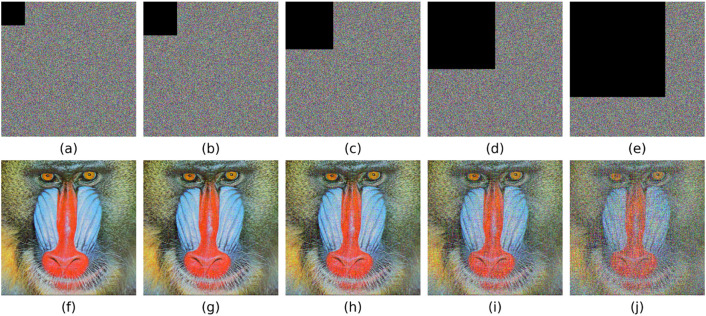
Robustness of the Encryption Algorithm Against Aata Loss. (a) ,(b),(c),(d),(e) Cipher Image with 1/32, 1/16, 1/8, 1/4, and 1/2 of the Information Lost; (f) ,(g),(h),(i),(j) Decrypted Image of (a) ,(b),(c),(d),(e).

In addition to noise pollution, data loss may also occur in encrypted images during application. This requires that the image can still extract as much original information as possible after being cropped to a certain extent. To verify the algorithm’s ability to resist cropping attacks, we cropped the upper-left 1/32, 1/16, 1/8, 1/4, and 1/2 regions of the cipher image of Baboon, and then decrypted the cipher image with some cipher pixels lost. The experimental results are shown in Fig 14. And Table 10 shows that even when subjected to attacks of different degrees, the decrypted image can still maintain high PSNR and SSIM, indicating that the proposed algorithm possesses strong resilience to data corruption.

**Table 10:** PNSR and SSIM under different cropping attacks.

Image		Cropping ratio				
		1/32	1/16	1/8	1/4	1/2
Baboon	PSNR	23.88	20.82	17.81	14.83	11.82
	SSIM	0.8996	0.8134	0.6800	0.4956	0.2695
[53]	PSNR	——	19.67	——	13.99	——

### 5.9. Resistance to chosen plain attacks

**Fig 15:**
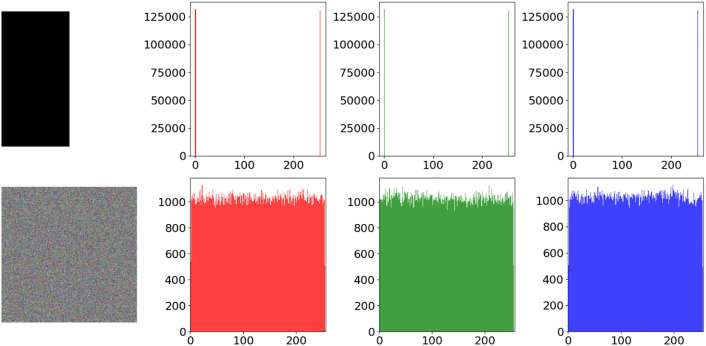
Half black and half white image encryption effect.

To verify the algorithm’s ability to resist chosen-plaintext attacks, this study selects 512 × 512 half-black and half-white images for encryption tests. As shown in Fig 15, the encrypted image are not only completely unrecognizable, but their pixel histograms also exhibit an ideal uniform distribution. Experimental results demonstrate that the algorithm is capable of resisting chosen-plaintext attacks even under extreme conditions, without compromising the reliability of the encryption and decryption processes when confronted with special images.

## 6. Conclusion

To address the issues of insufficient performance in classical chaotic maps and the complex structure of novel chaotic maps, this paper proposes a new two-dimensional simplified quadratic sine map (2D-SQSM). Multi-dimensional assessments, including Lyapunov exponents, sample entropy, permutation entropy, and NIST tests, show that 2D-SQSM outperforms existing advanced chaotic maps in terms of chaotic performance. Utilizing this basis, we implemented a color image encryption algorithm. In the scrambling stage, first perform row-column permutation and transpose operations on the RGB channels respectively, and then deeply scramble the pixel positions of the comprehensive matrix through cyclic shifting; In the diffusion stage, perform an XOR operation on the chaotic sequence generated by 2D-SQSM and the image pixels, significantly enhancing the encryption randomness. The experimental results demonstrate that this algorithm significantly enhances the image information entropy, reduces pixel correlation, and has strong resistance to common attack types like differential attacks, noise attacks, cropping attacks, and chosen plaintext attacks. This implies that the algorithm is robust and suitable for scenarios of secure transmission. In addition, through analysis, the complexity of the encryption algorithm proposed in this paper in terms of both time and space is O(N), which indicates that this algorithm is efficient in terms of complexity and suitable for processing large-size images. Although the encryption algorithm cascade structure proposed in this paper is simple to implement and has clear logic, and the scrambling and diffusion stages are relatively independent, there may theoretically be a risk of being attacked step by step. Future work will explore a more tightly coupled encryption structure to further enhance security.
